# A Case Report: Long Post-COVID Vaccination Syndrome During the Eleven Months After the Third Moderna Dose

**DOI:** 10.7759/cureus.32433

**Published:** 2022-12-12

**Authors:** Josef Finsterer

**Affiliations:** 1 Neurology, Neurology and Neurophysiology Center, Vienna, AUT

**Keywords:** neuro-cognition, adverse reaction, side effects, sars-cov-2 vaccination, long post-covid vaccination syndrome

## Abstract

It is undisputed that anti-SARS-CoV-2 vaccines can have side effects. Long post-COVID vaccination syndrome (LPCVS) is one of them and is often neglected. It persists 11 months after the third mRNA-1273 (Moderna) vaccine dose has not been reported. Our patient is a 39-year-old male with a largely uneventful previous history who developed severe adverse reactions immediately after the third dose of the mRNA-1273 (Moderna) vaccine. In addition to brief fever, headache, flickering eyes, skin rashes, tiredness, disorientation, dizziness (brain fog), tiredness, impaired thinking and concentration, and emotional disorders occurred as a result. Cerebral MRI showed non-specific white matter lesions in a frontotemporal distribution. Some of the immune parameters were deflected. Non-steroidal anti-inflammatory drugs, antihistamines, sartans, and statins have occasionally provided temporary relief. In conclusion, LPCVS is a definite complication of anti-SARS-CoV-2 vaccinations and can severely impact the quality of life and lead to disability. Despite extensive work-up, a clear cause for the long-term neuro-cognitive deficits cannot be identified. Symptomatic treatment can provide some relief. Patients with LPCVS should be taken seriously and treated appropriately.

## Introduction

There is increasing evidence that SARS-CoV-2 vaccinations of any brand can be complicated by mild or severe and short or long-lasting adverse reactions [1]. In most cases, these side effects are mild and short-lived. However, in a number of patients adverse reactions to anti-SARS-CoV-2 vaccines can persist and be severe or even life-threatening and fatal [2]. If adverse reactions last longer than four weeks, one speaks of the long post-COVID vaccination syndrome (LPCVS) in analogy to the long-COVID syndrome [3,4]. Although the cause of LPCVS is unknown, it has been attributed by some groups to multisystem inflammatory syndrome (MIS) [5]. Long post-COVID vaccination syndrome persisting over 11 months after the third mRNA-1273 (Moderna) vaccine dose has not been previously reported.

## Case presentation

The patient is a 39-year-old male Swiss resident, height 183cm, weight 81kg, who tolerated the first dose of the mRNA-1273 (Moderna) vaccine in May 2021 without major side effects. After the second dose in June 2021, he had a fever of up to 39.5°C, drowsiness, a robotic feeling, and a rash on the lower limbs for five days. After the third dose, he developed a fever of up to 38.8°C on the day of the vaccination and a day later severe headache, flickering eyes, and tiredness one day later (acute phase). On the third day post-vaccination, he experienced drowsiness (brain fog), de-realization, and lack of imagination. His thinking ability was impaired, his emotions were gone, and his hands became numb at night. After closing his eyes, he had no ideas, no conceptions, and no memories. Any attempt to regain imagination after the eyes were closed was met with a certain resistance. His symptoms of LPCVS are listed in Table 1. 

**Table 1:** Symptoms of LPCVS, their frequency, course, treatment, and outcome. For symptoms where the onset is unknown, it is likely that more severe symptoms overlapped the recognition or memory of the symptom. This is especially true in the very first days when the patient primarily required permanent bed rest. Symptoms and their intensity were commonly random with a base affectedness. Nonsteroidal antirheumatic drugs (NSAR) resolved the brain fog/numbness for approximately five days after intake but symptoms reoccurred within one to three days. Each treatment initially worsened symptoms followed by temporary relief of the symptoms once the medications were stopped until they reoccurred. Four attempts were made over two months using different NSARs (aspirin 3x300mg for 3.5 days and then another two days; naproxen 2x600mg for 1.5 days; ibuprofen 3x600mg for five days). A later attempt with ibuprofen (2x200mg) caused strong tearing symptoms in the left head. The same holds for oral methyl-prednisolone (4mg/d). Varying body temperature has been observed already after the second vaccination lasting two to three weeks. The same applied to the strong skin reaction between the legs. The patient is currently still taking drugs. In October 2022, two days of absence of statins induced a feeling as if the head would tear apart yielding to a complete inability to meet daily requirements. N/A: Not available, NSAR: Non-steroidal anti-rheumatic drugs, QoL: Quality of life, VAS: Visual analog scale, LPCVS: Long post-COVID vaccination syndrome

Symptom	Onset after 3^rd^ dose	Duration of symptom	Frequency	Course	Treatment	Resolution state	Resolution date	Impact on QoL	Impact on work
Partially extreme headache (up to VAS 8)	1 day	24 hours	once	exacerbating	painkillers	resolved	1/2022	high	high
De-realization	1-2 days	24 hours	every day	undulating	NSAR, the remainder was resolved by sartans, statins	resolved	4/5, 6/7 2022	high	high
Brain fog (extreme numbness)	1-2 days	24 hours	every day	undulating	N/A	resolved	16.2.2022 (?)	high	high
Cognitive impairment (as if packed in a cocoon)	1-2 days	24 hours	every day	undulating	NSAR	resolved	4/5 2022	high	high
Pulling, left temporal pressure, dragging on the left part of the head (correlated with cognitive impairment during first 7 months) (see figure 2)	~1-2 days (?), changed over time	24 hours	every day	undulating	various drugs relieved it for several hours, fluvoxamine was fading it out, but cognitive impairment remained	unresolved	N/A	high	now medium, (initially high)
Grogginess	< 2 days	24 hours	every day	undulating	NSAR, sartans, statins	partially resolved, rare now	4/5/8 2022	high	high
Stitching on the scalp	at day 2	<1s	once, at night, after relief of a severe headache	monophasic	N/A	N/A	N/A	N/A	N/A
Undulating body temperature (+-0.5 degrees)	3-4 days after the acute phase	2-3 weeks	every day	undulating	N/A	resolved	end of January 2022 (?)	low	N/A
Pain in the right armpit (vaccination arm)	~ several days	several hours	randomly every few days	monophasic	N/A	resolved	6/2022 (?)	low	N/A
Pressure on forehead	~ several days	occasionally 24 hours	several days or longer	monophasic	NSAR, statins	resolved	6/7 2022	high	medium
Painful right armpit/axilla	several days	several hours	several times a week	monophasic	N/A	resolved	4/5 2022	low	N/A
Strongly impaired cognitive functions and concentration, lack of abstraction ability	2-3 days after the acute phase	several hours to permanently	continuously over several months	undulating	NSAR highly beneficial	partially resolved	4/5 2022, ongoing with reduced severity	high	high
Loss of imagination (darkness without any imagination after eye closure; thoughts canceled by a stabbing pain in the head)	~ several days after the acute phase of the vaccine	permanently	permanently over several months	monophasic	NSAR	resolved	4/5 2022	high	medium
Polyarthralgia	<1 week	several hours	several times	exacerbating	NSAR beneficial temporarily (aspirin 3x300mg aspirin, 2x600mg naproxen, 3x600mg ibuprofen)	partially resolved, more rarely	5/6 2022 (?)	medium	N/A
Feeling like an anchor at random points in the brain that pulls randomly in all directions	<1 week	several times in a supine position	several weeks/months, randomly	monophasic	likely NSAR	resolved	4/2022 (?)	high	medium
Swaying feeling	<1 week	< several hours after 3^rd^ dose	almost every day when going to sleep	monophasic	N/A	unresolved	N/A	medium	N/A
Absence of dreams	within 1 week	several hours	every day	monophasic	N/A	resolved	N/A (likely 4/5 2022 onwards)	high	N/A
Feeling of heat in the head	within 1 week	several hours	randomly almost every day	exacerbating	N/A	resolved	3/2022 (?)	N/A	N/A
Pulling/shaking/pulsating feeling inside the head at night	within 1 week	< several hours	every day when going to sleep	monophasic	N/A	unresolved	N/A	medium	N/A
Insomnia	within 1 week	1-2 months (?)	every day	monophasic	N/A	resolved	2/2022	Low	N/A
Swelling in the neck (see Figure 1)	15.1.2022	~2-3 weeks	once in this area	exacerbating	N/A	resolved	6.2.2022	low	N/A
Slight swelling at the left temple/thickened red skin/slight bumps	several days or weeks	1-2 days	several times every few weeks	monophasic	H1 or H2 blockers	partially resolved	N/A	low	N/A
Staggering vertigo/dizziness	~ 1-2 weeks	several hours	almost every day	undulating	NSAR	resolved	4/2022 (?)	high	high
White light after eye closure and sleeping/half-sleeping	~ 1-2 weeks	<1s	randomly, every few days	undulating	N/A	unresolved, less frequent	N/A	low	N/A
Light persistence after eye closure and when eyes are exposed to light/shadow	~ 1-2 weeks	20s-60s	randomly, depending on light/shadow	undulating	after aspirin 900mg, naproxen, and ibuprofen less often, less comprehensive, shorter	partially resolved	4/5 2022	low	N/A
Blue or white spots after eye closure during the day	~ 1-2 weeks	<10s	every few days	monophasic	N/A	unresolved, less frequent	N/A	low	N/A
Whole body vibration	~ 1-2 weeks	several times in the supine position	several weeks, randomly	monophasic	N/A	resolved	2/3 2022 (?)	low	N/A
Panic attacks	~ 1-2 weeks	<0.5 hours	randomly every few days, often at night	exacerbating	NSAR	resolved	4/5 2022	high	N/A
Pain in the left ear	~ 1-2 weeks	<1 hour	randomly every few days	undulating	N/A	resolved	5/2022 (?)	N/A	N/A
Word finding disorders	~ 2 weeks	N/A	several times a day	monophasic	N/A	resolved	N/A	high	high
Right gonalgia	several weeks (reduced motion at the beginning)	N/A	while utilizing	exacerbating	N/A	largely resolved, depends on knee load	9/10 2022 (?)	low	N/A
Large skin erythema between legs	14.2.2022	permanently	only once	undulating intensity, the base symptom was monophasic	steroids and fungal cream for 1 to 2 weeks converted it to a red ring of slight uprisings after about 2 to 3 months	largely resolved, light brown area remaining	11.10.2022 (~)	N/A N/A	N/A
“Empty"/“cold” head feeling	March/April 2022	<1 hour	almost every day in the morning	monophasic	N/A, probably statins, sartans, or time	largely resolved	N/A	high	N/A
Pinch feeling inside left forehead/narcotic feeling left forehead/periorbitally	Apr / May 2022	several hours	randomly	monophasic	NSAR with residual symptom	largely resolved, the "pinch feeling” re-occurred (11/2022)	6/2022 (?)	medium	low
Tension headache	several weeks or months after the vaccination	sometimes several hours during the day, often at night	once every week or every two weeks	exacerbating	H1 or H2 blockers	N/A	N/A	low	low
Various small skin reactions on the legs	20.6.2022	1-2 days (?)	only once	unknown	N/A	resolved	22.6.2022	N/A	N/A
Delayed shadows after eye closure	~ 6 months	<1 second	on occurrence, once every few days when going to sleep	monophasic	N/A	unknown	N/A	medium	N/A
Numbness of digits 3 to 5 of the right hand during the night	27.7.2022	< several hours	a few days in a row, generally rarely	monophasic	N/A	partially resolved, less often	N/A	low	N/A
Fasciculations	approximately August 2022	<10 seconds	every few days	monophasic	triggered by statins	unresolved	N/A	N/A	N/A
Mild lymph node swelling on left armpit	4.9.2022	1-2 days	only once	exacerbating	N/A	resolved	5.9.2022	low	N/A
Twitches of the left upper lid	October 2022	<5s	every few days, occasionally 3 days in a row	undulating	N/A	largely resolved	10.11.2022	low	N/A
Disturbed ejaculation	unknown	on occurrence	on occurrence	on occurrence	N/A	resolved	4/5 2022 (?)	low	N/A
Slight flickering in the eyes/vision particularly after waking up	unknown	<0.5 hours	almost every day	monophasic	N/A	N/A	N/A	medium	N/A

**Figure 1:**
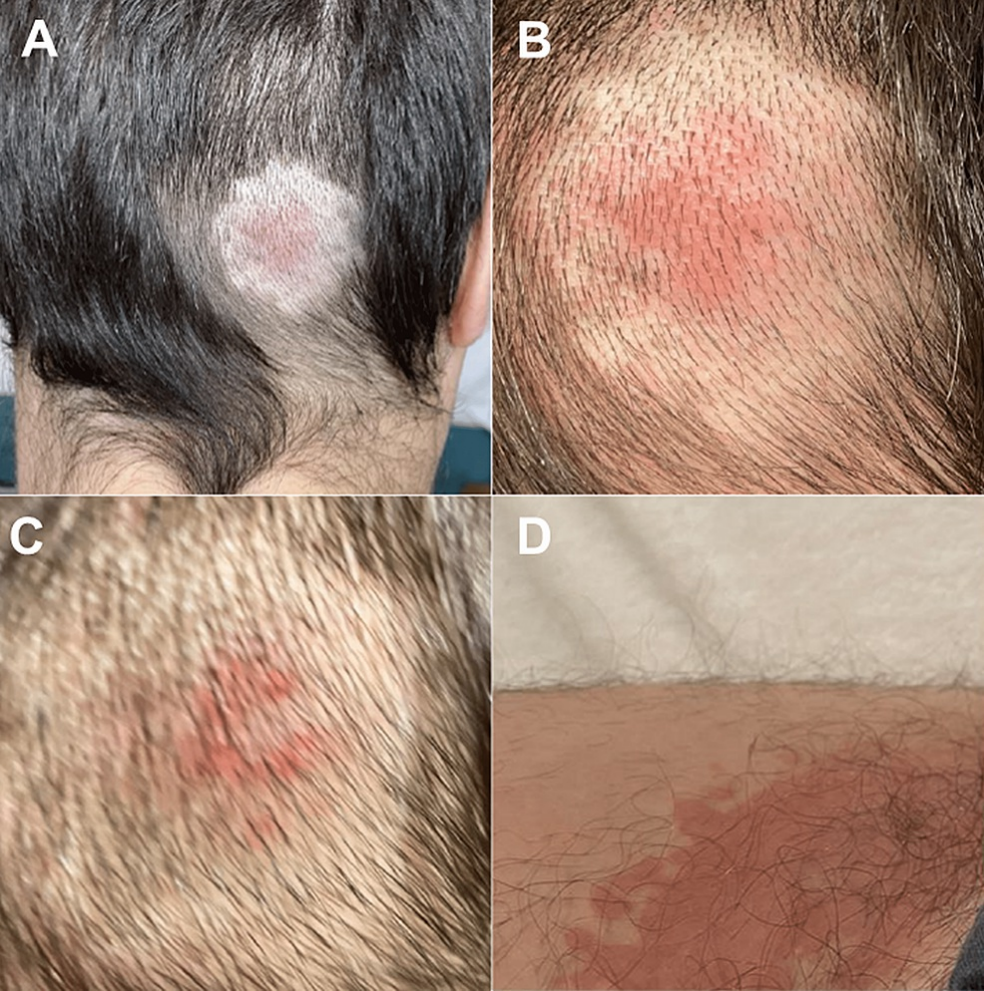
Skin rash six days after the third dose A, B & C: Fading raised redding on the right occipital after the third Moderna dose, D: Tinea inguinalis

**Figure 2:**
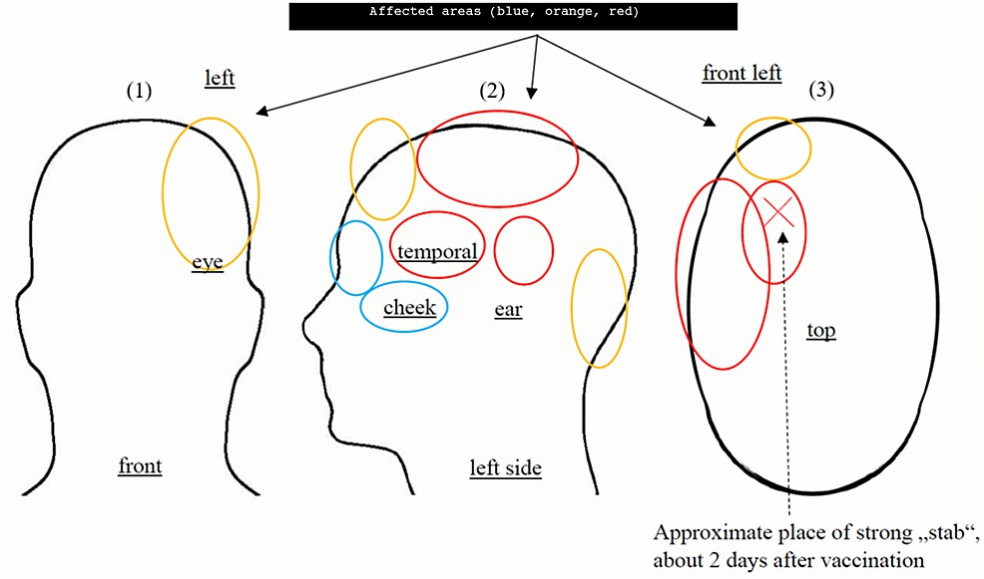
Position of tension/tearing feeling that causes dizziness and partially impaired cognitive capabilities Symptoms move within these areas in partially <15 minutes and partial relief overnight. Symptoms are partially but not necessarily location-dependent (panels (1) and (2)). The extracranial disturbances might proxy the processes intracranially (red: tension causes strong impairment; orange: tension causes moderate or no impairment; blue: no impairment). The X depicts the approximate position of the stab feeling two days after vaccination (panel (3)). The red area induces symptoms that feel like a strong pressure pushing top-down onto this part of the head.

On the fourth day after the third dose, he developed bilateral tinea inguinalis. On the sixth day after the vaccination, he also developed a reddish occipital swelling (Figure 1). Two weeks post-vaccination, he developed disabling pulling in his head and pressure on his left skull (Figure 2). Additionally, he developed easy fatigability and impaired concentration accompanied by a temporary inability to grasp his own writings cognitively. Since the vaccination, there has also been right gonalgia under load (as seen in Table 1). There was a slight improvement in symptoms three weeks after the vaccination, but when he attempted to work at his original job, he had to call in sick repeatedly due to brain fog, disorientation, difficulty concentrating, and impaired abstract thinking. He also noted photophobia when exposed to intense light and high sensitivity to noise. Occasionally, while half asleep, he saw surreal, abstract images and reported palinopsia. There was also repetitive pulling, particularly over the left side of the head (Figure 2). Photophobia and brain fog improved after four months of non-steroidal anti-inflammatory drugs (NSAIDs) while it took another four months to fully recover cognitive functions. Since then, symptoms have occasionally re-appeared. His history was uneventful except for chronic sinusitis for 15 years, neurodermatitis for two years, keratoconus, and recurrent mild tinnitus that worsened after vaccination. He did not take any medication regularly.

**Figure 3:**
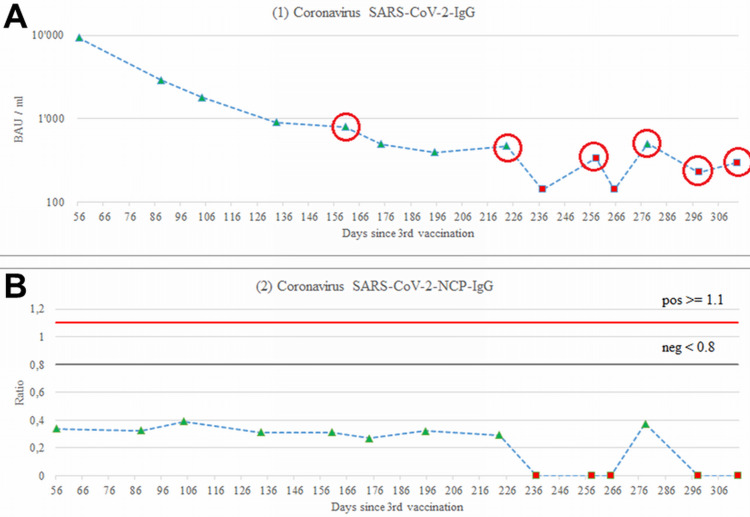
Spike antibody levels over time based on the drawing date as opposed to the vaccination date A: Normal NCP-IgG antibodies B: Red data points indicate results from a different laboratory than the green data points (both used the ELISA method). Yet, even laboratory values suggest inverting slopes. For instance, laboratory 1 reported a value of 394 (24.7.2022) and 505 (15.10.2022), an increase of over 28% despite 83 days of time decay. Likewise, laboratory 2 reported 142 (4.9.2022) and 226 (4.11.2022), an increase of 59% despite 61 days of time decay. Values in between highly fluctuate beyond 100% around these low absolute threshold areas. Measurements were re-assessed once by the laboratory suggesting a variation < +-11%. This is surprising as the case had no virus infection over time (lower panel). The red dots for the NCP are of qualitative nature from laboratory 2. NCP: Novel coronavirus pneumonia

The clinical-neurological examination was unremarkable. The C-reactive protein was 12 (n, 0-5mg/L). The blood sedimentation rate was 1 after the first hour. Blood cell counts were normal except for occasional monocytosis. Electrolytes, kidney, blood coagulation, and liver function parameters were repeatedly normal. The antinuclear antibody (ANA) and antineutrophil cytoplasmic antibodies (ANCA) were negative. Connective tissue disease screening including U1 small nuclear ribonucleoprotein particle (U1-snRNP), Sjögren's syndrome-related antigen A autoantibodies (SS-A/Ro), Sjögren's syndrome type B (SS-B/La), centromere protein B (CENP-B), topoisomerase 1 (Scl-70), anti-histidyl transfer RNA [t-RNA] synthetase) (Jo-1), Sm, anti-double-stranded deoxyribonucleic acid (dsDNA), fibrillarin, RNA Pol-III, ribosomal P proteins (Rib-P), overlap syndrome of polymyositis and scleroderma (PM-Scl), proliferating cell nuclear antigen (PCNA), and Mi2 was not informative. Cardiolipin antibodies, lupus anticoagulants, and beta-2 glycoprotein were within normal limits. Testing for autoantibodies showed positivity for b1-autoantibodies (aab), a1-aab, ET-aab, and b2-aab. Lymphocyte typing revealed normal counts for the cluster of differentiation (CD)3, C16/56, CD4, CD8 T-lymphocytes, normal counts for CD19 B-lymphocytes, and normal total lymphocytes. The CD4/CD8 ratio was also reduced, as was the relative number of CD4 T-lymphocytes. The relative number of CD8 cells was increased. Unstimulated interleukins (IL-10, IL-12, IL-17 IL-1b, IL-2, IL-4, IL-6, IL-8 TNF-a) were within normal limits but pro-inflammatory cytokines IL-6 and IL-1β were reduced and IL-8 elevated. Antibodies against the spike protein were repeatedly increased during the disease course but continuously decreased (Figure 3). Antibodies against the N-protein were normal, indicating no prior SARS-CoV-2 infection. A second determination of interleukins revealed increased tumor necrosis factor-alpha (TNF-a), vascular endothelial growth factor (VEGF), interferon (IFN)-g, IL-6, IL-8, and IL-10. Vitamin levels were within normal limits, with the exception of low vitamin B12 and vitamin C. Salivary cortisol levels were elevated at baseline and after five and eight hours.

**Figure 4:**
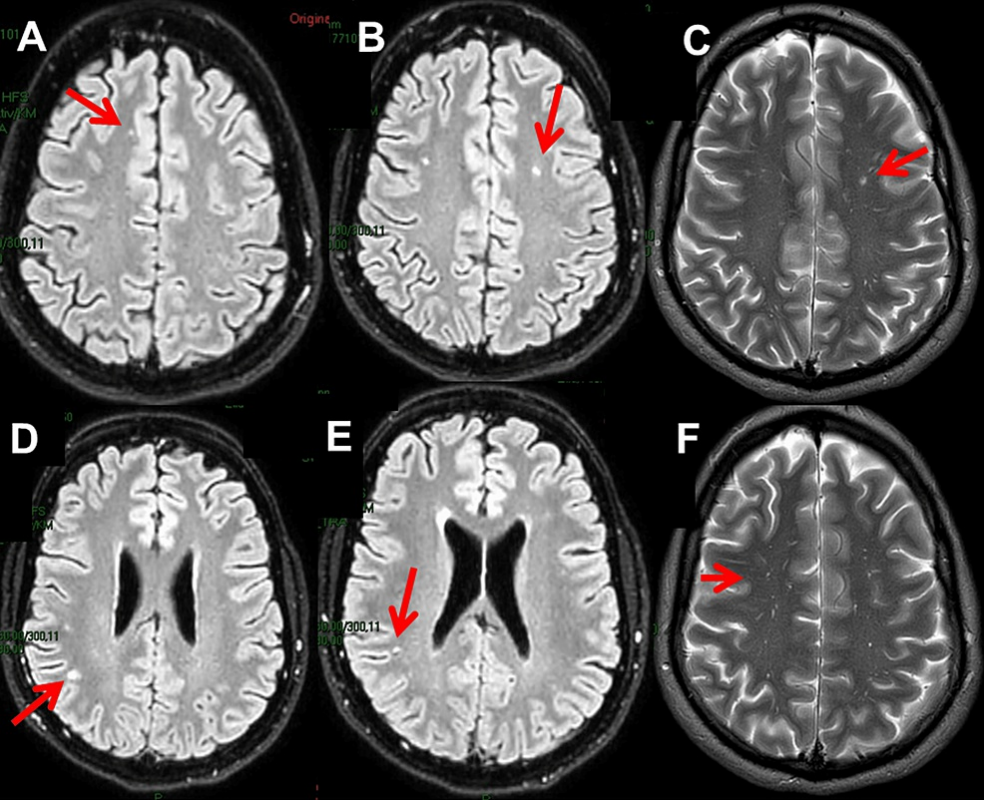
Cerebral MRI two months after the onset of LPCVS showing spot-like T2 (A, B, D, E) and FLAIR (C, F) hyperintensities in a frontotemporal distribution LPCVS: Long post-COVID vaccination syndrome, FLAIR: Fluid attenuated inversion recovery

Cerebral magnetic resonance imaging (MRI) seven weeks after vaccination showed nonspecific T2 and fluid-attenuated inversion recovery (FLAIR)-hyperintense white matter lesions (WMLs) in a frontoparietal distribution and polypoid mucosal swelling with retention cysts in all paranasal sinuses (Figure 4). The carotid ultrasound was non-informative. Electroencephalography (EEG) six weeks after vaccination showed only discrete theta waves over the left frontotemporal projections. Despite an initial recommendation, the patient decided not to have cerebrospinal fluid (CSF) investigations due to his own risk-benefit considerations and due to the lack of treatment options on the part of the treating physicians. The ECG and transthoracic echocardiography were normal. The patient noted that NSAIDs only resulted in a temporary improvement in brain fog and cognitive functions at first (Table 1). He also experienced positive effects from antihistamines, sartans, and statins taken as needed. In particular, sartans and statins improved cognitive dysfunction and resulted in symptom stability. Antihistamines particularly reduced hypersensitivity. Nattokinase, quercetin, and FibroProtek® were taken as needed and occasionally showed some relief. A single dose of methyl-prednisolone worsened the symptoms.

## Discussion

This case shows that SARS-CoV-2 vaccinations can cause severe side effects that are long-lasting (LPCVS) and can severely limit the quality of life and lead to disability. The case also shows that these side effects can be objectified. WMLs and abnormal immunological parameters were the main abnormalities identified during the workup of the complaints. The case also shows that NSAIDs, antihistamines, sartans, and statins can have a beneficial effect, at least temporarily. Although these side effects primarily affected the brain, it was a multisystem reaction. In addition to the brain, the ears, joints, and skin were also involved in LPCVS.

Symptoms and signs were attributed to vaccination because they were absent prior to vaccination and because of the strong temporal association. Another argument for a causal relationship between the described abnormalities and vaccinations is that such long-term adverse reactions have been reported before [6,7]. Another argument for a causal relationship between COVID vaccines and LPVCS is that adverse reactions to the vaccines, such as myopericarditis, have been experimentally induced in animal models [8].

In general, the symptoms and signs of LPCVS are highly variable and can be associated with or without abnormal findings on instrumental examinations. Long post-COVID vaccination syndrome can occur at any age, in either sex, and with any vaccine brand [4]. Long-lasting side effects of SARS-CoV-2 vaccines generally include chills/fever, fatigue, lethargy, dizziness, headache, migraine, ageusia, anosmia, visual disturbances, syncope, palpitations, burning sensation, facial paralysis, parosmia, poor sleep quality, seizure, transient ischemic attack, tremor, thyroiditis, nausea, abdominal pain, diarrhea, vomiting, hypoesthesia, neuralgia, paresis, myalgia, muscle cramps, arthralgias, and various skin reactions [4,9,10,11]. One of the most common long-term side effects of SARS-CoV-2 vaccines, which has been described in several hundred patients, is vaccine-induced immune thrombotic thrombocytopenia (VITT) [12]. It is estimated that VITT occurs in 0.5-1/100000 recipients of vector-based vaccines from AstraZeneca and Johnson & Johnson [13]. Vaccine-induced immune thrombotic thrombocytopenia can be complicated by thrombosis or bleeding due to dysfunctional platelets [14]. The abundance of clinical responses to COVID vaccines previously reported is consistent with all manifestations of LPCVS in the index patients.

Several hypotheses have been put forward to explain the occurrence of adverse reactions in anti-SARS-CoV-2 vaccinations. The first hypothesis assumes that COVID vaccine side effects are due to MIS [5]. Arguments for such a mechanism are that it has been previously reported and that inflammatory and immunological markers can be elevated in COVID-vaccine patients. The index patient was excluded from MIS because he did not meet Brighton Collaboration Case Definition (BCCD) criteria. The second hypothesis is based on the assumption that COVID vaccines produce an allergic reaction. Arguments for this hypothesis are reports on vaccination-induced mast cell activation syndrome (MCAS) [15]. The skin lesions and the beneficial effect of antihistamines in the index patient support the hypothesis that allergenic mechanisms are involved in the development of side effects. Another argument for hypothesis two is reports of chronic, spontaneous urticaria (CSU) after SARS-CoV-2 vaccinations [16]. The probability of a CSU recurrence within three months after the Biontech-Pfizer vaccination correlates with a positive autologous serum skin test, allergic comorbidities, and basopenia [16]. A third theory suggests that the adverse reaction is triggered by the generation of the spike protein after vaccine injection in the host [17]. According to this hypothesis circulating S-protein subunits/peptide fragments not only activate the immune system but also bind to angiotensin-converting enzyme 2 (ACE2) receptors not only on endothelial cells but also on multiple cell types surrounding the capillary beds but due to antigen diffusion [17]. However, there is also evidence that lipid nanoparticles used for mRNA delivery induce a pro-inflammatory reaction [17]. A fourth hypothesis suggests that COVID vaccines suppress the immune response via G-quadruplexes, exosomes, and microRNAs [18]. An argument in favor of this hypothesis is supported by the fact that there is also evidence that SARS-CoV-2 vaccinations can reduce immune competence and that superinfections can follow [19]. Another argument for hypothesis four is that there is evidence that SARS-CoV-2 vaccination can trigger flairs of pre-existing immunological disease or can even induce a new immunologic disease.

## Conclusions

Long post-COVID vaccination syndrome is a definite complication of anti-SARS-CoV-2 vaccinations and can severely impact the quality of life and lead to disability. Despite extensive workup, a clear cause for the long-term neuro-cognitive deficits may not be identified. However, symptomatic treatment can provide relief. Patients with LPCVS should be taken seriously and treated appropriately.
